# Insights into Terminal Sterilization Processes of Nanoparticles for Biomedical Applications

**DOI:** 10.3390/molecules26072068

**Published:** 2021-04-03

**Authors:** Sergio A. Bernal-Chávez, María Luisa Del Prado-Audelo, Isaac H. Caballero-Florán, David M. Giraldo-Gomez, Gabriela Figueroa-Gonzalez, Octavio D. Reyes-Hernandez, Manuel González-Del Carmen, Maykel González-Torres, Hernán Cortés, Gerardo Leyva-Gómez

**Affiliations:** 1 Departamento de Farmacia, Facultad de Química, Universidad Nacional Autónoma de México, Ciudad de México 04510, Mexico; q901108@hotmail.com (S.A.B.-C.); luisa.delpradoa@gmail.com (M.L.D.P.-A.); hiram.qfohead@gmail.com (I.H.C.-F.); 2 Escuela de Ingeniería y Ciencias, Departamento de Bioingeniería, Tecnológico de Monterrey Campus Ciudad de México, Ciudad de México 14380, Mexico; 3 Departamento de Fisiología, Biofísica & Neurociencias, Centro de Investigación y de Estudios Avanzados del Instituto Politécnico Nacional, Ciudad de México 07360, Mexico; 4 Departamento de Biología Celular y Tisular, Facultad de Medicina, Universidad Nacional Autónoma de México, Ciudad de México 04510, Mexico; davidgiraldo@comunidad.unam.mx; 5 Unidad de Microscopía, Facultad de Medicina, Universidad Nacional Autónoma de México, Ciudad de México 04510, Mexico; 6 Laboratorio de Farmacogenética, UMIEZ, Facultad de Estudios Superiores Zaragoza, Universidad Nacional Autónoma de México, Ciudad de Mexico 09230, Mexico; gabriela.figueroa@zaragoza.unam.mx; 7 Laboratorio de Biología Molecular del Cáncer, UMIEZ, Facultad de Estudios Superiores Zaragoza, Universidad Nacional Autónoma de México, Ciudad de México 09230, Mexico; octavio.reyes@zaragoza.unam.mx; 8 Facultad de Medicina, Universidad Veracruzana, Mendoza 94740, VER, Mexico; manugonzalez@uv.mx; 9 CONACyT-Laboratorio de Biotecnología, Instituto Nacional de Rehabilitación Luis Guillermo Ibarra Ibarra, Ciudad de México 14389, Mexico; mikegcu@gmail.com; 10 Laboratorio de Medicina Genómica, Departamento de Genética, Instituto Nacional de Rehabilitación-Luis Guillermo Ibarra Ibarra, Ciudad de México 14389, Mexico

**Keywords:** sterilization, nanoparticles, autoclaving, filtration, ionizing radiation, nonionizing radiation

## Abstract

Nanoparticles possess a huge potential to be employed in numerous biomedical purposes; their applications may include drug delivery systems, gene therapy, and tissue engineering. However, the in vivo use in biomedical applications requires that nanoparticles exhibit sterility. Thus, diverse sterilization techniques have been developed to remove or destroy microbial contamination. The main sterilization methods include sterile filtration, autoclaving, ionizing radiation, and nonionizing radiation. Nonetheless, the sterilization processes can alter the stability, zeta potential, average particle size, and polydispersity index of diverse types of nanoparticles, depending on their composition. Thus, these methods may produce unwanted effects on the nanoparticles’ characteristics, affecting their safety and efficacy. Moreover, each sterilization method possesses advantages and drawbacks; thus, the suitable method’s choice depends on diverse factors such as the formulation’s characteristics, batch volume, available methods, and desired application. In this article, we describe the current sterilization methods of nanoparticles. Moreover, we discuss the advantages and drawbacks of these methods, pointing out the changes in nanoparticles’ biological and physicochemical characteristics after sterilization. Our main objective was to offer a comprehensive overview of terminal sterilization processes of nanoparticles for biomedical applications.

## 1. Introduction

According to the Internationational Standardization Organization and the IUPAC, nanoparticles are defined as particles with sizes ranging between 1 and 100 nm [1]. These systems may be elaborated from many natural and synthetic materials; thus, they can be designed to exhibit a wide range of biological and physicochemical properties that confer to them an enormous potential to be employed in numerous biomedical purposes [2]. Likewise, nanoparticles may be loaded with diverse molecules by dissolution, entrapment, absorption, or encapsulation. Therefore, their applications may include drug delivery systems, gene therapy, tissue engineering, photodynamic cancer therapy, and contrast agents in imaging [3,4,5,6]. These applications need nanoparticles to exhibit biocompatibility, low toxicity, nonimmunogenicity, and sterility.

Sterility is defined as the nonexistance of a viable microbe that could be hazardous to health. The primary contaminating agents are bacteria and fungi, and the contamination sources include employed materials, equipment, and operational personnel. Since nanoparticles sterility is an indispensable requisite for their in vivo use in biomedical applications, diverse sterilization techniques have been developed to remove or destroy microbial contaminations [7,8].

Currently, the main sterilization methods to sterilize nanoparticles are sterile filtration, autoclaving, ionizing radiation, and nonionizing radiation [9,10,11,12]; however, other utilized methods include treatment with chemical substances such as gas plasma, ethylene oxide, and formaldehyde. It is noteworthy that these methods may produce unwanted effects on the nanoparticles’ characteristics, affecting their safety and efficacy. For instance, the autoclaving process can alter the stability, zeta potential, average particle size, and polydispersity index (PDI) of diverse types of nanoparticles, depending on their raw materials [13]. Likewise, ionizing and nonionizing radiations may affect the stabilizing materials located on the surfaces of nanoparticles, which could modify the release profiles of drugs [14]. Therefore, the suitable method’s choice depends on diverse factors such as the formulation’s characteristics, batch volume, available methods, and desired application.

In this article, we describe the main sterilization methods of nanoparticles. Moreover, we discuss the advantages and drawbacks of these methods, pointing out the changes in nanoparticles’ biological and physicochemical characteristics after sterilization. Our main objective was to offer a comprehensive overview of the terminal sterilization processes of nanoparticles for biomedical applications.

## 2. Sterile Filtration

### 2.1. Fundament

**Figure 1:**
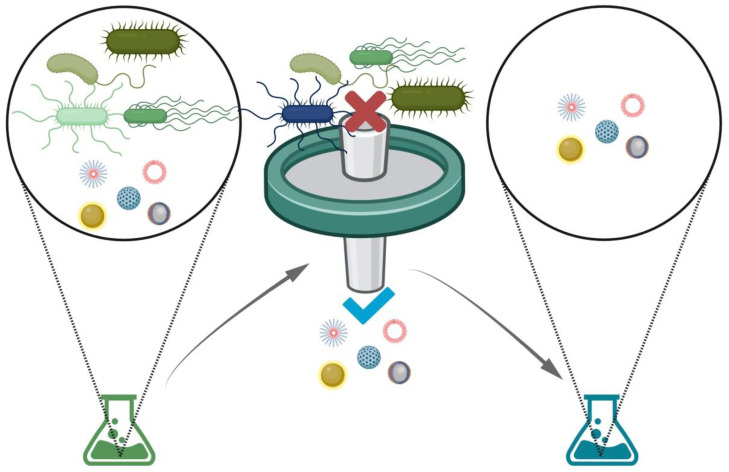
Schematic representation of the sterile filtration method. Contaminating agents such as bacteria or fungi could be present in nanoparticles; the filtration process may remove these pathogens and obtain sterile nanoformulations. Created with BioRender.com.

Sterile filtration is a sterilization technique to remove microorganisms from liquid nanoformulations (Figure 1). Membrane filters with pore sizes usually ranging between 0.2 to 0.45 μm are employed for this purpose [3,9,15,16]; nonetheless, filters with smaller pore sizes are commercially available. Therefore, filtered nanoparticles’ retention and elution behaviors will depend on their dimensions. Sterile filtration represents a feasible approach for the terminal sterilization of many types of nanosystems sensitive to heat or chemical substances, because, generally, it does not produce adverse effects on nanoparticles, and it does not generate toxic impurities. However, this method may exhibit some limitations related to formulations’ viscosity, filter clogging, or even structural integrity alterations. For example, low-viscosity nanoformulations with small sizes easily could pass through 0.2-μm membrane filters; however, this procedure could alter the structural integrity of bigger nanoparticles and produce loaded drug loss. Thus, the suitability of this method should be evaluated in each particular case.

### 2.2. Applications

Several research groups have suggested sterile filtration as a feasible method to sterilize nanoparticles without producing substantial adverse effects. For example, in a pioneering study, Masson et al. [9] investigated the impact of filtration with 0.2-μm membrane filters on poly-(ε-caprolactone) (PCL) nanospheres (size: 130–180 nm). According to their results, nanoparticles were efficaciously sterilized, and no aggregation and no effects on their morphology were observed. Another study evaluated the effects of filtration on PEGylated poly(γ-benzyl-l-glutamate) nanoparticle properties [15]. In this case, the authors used 0.22-μm membrane filters and measured the nanoparticle size, zeta potential, and PDI. Filtration produced a slight change in the zeta potential but did not change the size or PDI. Furthermore, no detectable microbial contamination was found after filtration. Therefore, the method was effective in obtaining sterile nanoparticles without significant physicochemical modifications. Very similar results were reported by Konan et al. [3] with polyester nanoparticles. These authors used 0.22-μm filters with modified polyethersulfone surfaces to sterilize their nanoformulation. They did not find significant changes in the nanoparticle sizes and PDI. Moreover, sterility testing demonstrated no bacterial or fungi contamination, indicating this sterilization method’s suitability for these nanoparticles. Likewise, another study showed that amphotericin B-loaded liposomes larger than 200 nm could be sterilized through filtration with 0.2-μm membrane filters without undergoing significant alterations in their shape, size, and physicochemical properties [17].

Although those studies demonstrated the efficacy of filtration for the terminal sterilization of different nanosystems, other studies have shown contrasting results. For example, Li and Deng [18] reported that when liposomes contain aqueous encapsulated compounds, these can leak when liposomes pass through membrane filters. Likewise, Bos et al. described similar findings in poly(2-(dimethylamino) ethyl methacrylate)-based gene transfer complexes [19]. The authors found that filtration did not affect the loaded DNA integrity, but its concentration was decreased.

Finally, concerning industrial applications, some companies have patented nanoformulations that employ filtration for their terminal sterilization. For example, Cerulean Pharma Inc. patented diverse cyclodextrin-based nanoformulations intended to deliver therapeutic agents [20]. Similarly, Janssen Biotech Inc. patented a pharmaceutical formulation consisting of a nanoemulsion containing propofol [21]. According to its statement, the formulation remained chemically and physically stable after filtration through a Polyvinylidene Fluoride filter with a 0.2-μm pore size.

### 2.3. Advantages and Disadvantages

One of the main advantages of sterile filtration is that it may be applied to nanoformulations loaded with compounds that could be denatured by radiation or heat. Likewise, in many cases, the sterilization method can eliminate microbial contamination from nanoparticles without modifying their physicochemical properties and functionality. Finally, the filtration’s other potential advantages are the nanoparticle solution’s recyclability and relatively easy scale-up [22].

Despite these remarkable advantages, the utilization of these filters make the sterilization of large nanoparticles difficult. For example, it is not usually viable to apply sterile filtration to magnetic particles, because their sizes range over 200 nm, a bigger size than the pore diameter of membrane filters that effectively remove microorganisms [23]. Likewise, nanocarriers with sizes larger than 200 nm can obstruct the pores, causing the loss of particles and leading to a decreased yield after filtration [16,24,25]. On the other hand, filtration also could affect the structural integrity of specific nanoparticles. A recent study demonstrated that filtration decreased the hydrodynamic size of dextran-coated magnetic iron oxide nanoparticles (from 152.7 nm to 131.6 nm), altering their dextran/iron oxide proportion significantly [11]. Depending on the wanted purpose of the nanoparticles, this result might have adverse effects on some attributes, such as drug bioavailability, body distribution, or therapeutic activity. Thus, this sterilization procedure could be inadequate for certain types of nanoparticles, regardless of their sizes.

In summary, filtration sterilization may be ideally used to sterilize heat- or radiation-sensitive nanoparticles. Moreover, due to the filter’s pore size, nanoparticles with a size distribution smaller than 220 nm can be sterilized by this method. However, formulations with bigger sizes, high viscosities, or high solids concentration will be difficult to filter. A filter obstruction could occur in these cases, leading to a reduced nanoparticle recovery [24]. Finally, although this method effectively eliminates microorganisms, it does not remove pyrogens or endotoxins; thus, it is necessary to ensure aseptic manufacturing conditions and reagent purity in elaborating nanoparticles [26].

## 3. Autoclaving

### 3.1. Fundament

Moist sterilization or autoclaving is an effective method accepted by international norms that exposes the nanoparticles at high-pressurized steam at around 120 °C for 15–20 min. The steam in the autoclave can penetrate freely through the materials, and sterilization is easily achieved [8,27,28]. The autoclaving can be configurated by steps of increasing temperatures at the water boiling point in a lapse of at least three days without pressurization; this variation is known as Tyndallization [29]. The autoclaving effectively inactivates bacteria, viruses, and other biological materials; thus, this method is suitable for the terminal sterilization of diverse formulations.

### 3.2. Applications

Metal nanoparticles do not present a significant alteration in their physical properties, such as size or morphology, after autoclaving [8,11,28,30,31,32]. However, nanoparticle aggregation and formulation stability after autoclaving depend on the capping materials [8,28]. For example, gold nanoparticles capped with tiopronin presented a slight increase in size, from 2 nm to 5 nm, and agglomerate after autoclaving. The agglomeration and the increased size are due to the growth and recrystallization processes (Ostwald ripening) induced by the autoclaving temperatures. The same formulation presented a color change from brown to reddish. This color change is interpreted as a different surface plasmon resonance band on a UV-Visible spectra scanning analysis. Interestingly, these changes were not present in a formulation where the gold nanoparticles were capped with poly(ethylene glycol) (PEG) [8].

Similarly, lipid-based nanoparticle formulations also have acceptable performance after autoclaving sterilization. For example, Mancini et al. [33] submitted a tripalmitin-based nanoparticle formulation to autoclaving (at 105 or 121 °C) and fractional sterilization (80 or 60 °C/30 min/3 days). This formulation was repeated using polyvinyl alcohol (PVA), sodium deoxycholate, or polysorbate-20 as surfactants. PVA protected the lipid core in the formulation and conserved the particle size around 200 nm in both sterilization processes. In another research, Hippalgoamkar et al. [10] evaluated the autoclaving effect in solid lipid nanoparticles loaded with Indomethacin. The nanoparticles were stabilized with polysorbate-80 and sterilized at 110 °C for 30 min. The size, zeta-potential, pH, and percentage entrapment efficiency (EE) did not significantly differ from the initial values before the autoclaving.

On the other hand, the autoclaving process is suitable for nanoparticles based on materials as minerals. However, the synthesis method of the nanoparticles influences their performance in autoclaving sterilization. For example, Santos et al. [34] explored the sterilization effects by autoclaving on hydroxyapatite-based nanoparticles. Nanoparticles synthesized by hydrothermal (at 180 °C in an oven for 24 h) and wet chemical (at 37 °C in a thermostatic water bath for 24 h) procedures presented good stability after autoclaving at 120 °C for 20 min. The authors analyzed the nanoparticles’ morphology and sizes by transmission electron microscopy (TEM) and X-ray diffraction. The hydrothermally synthesized nanoparticles did not present size or visible morphology changes. In contrast, wet chemical synthesized nanoparticles presented thicker sizes and agglomerations with observable changes in morphology. Moreover, the X-ray diffraction analysis revealed that hydrothermally synthesized nanoparticles had higher crystallinity than wet chemical synthesized nanoparticles. These findings indicate a better performance from the hydrothermally synthesized nanoparticles after sterilization by autoclaving.

Hagbani et al. [35] designed an assemble curcumin complexation with cyclodextrins while the autoclaving process occurred. The thermostability of curcumin under autoclaving conditions was examined by ^1^H nuclear magnetic resonance (^1^H-NMR), Raman spectroscopy, differential scanning calorimetry (DSC), and X-ray powder diffraction (XDR). Interestingly, the curcumin cyclodextrin complex could entrap the curcumin efficiently after autoclaving. The curcumin exhibited specific peaks from 6.59 to 7.57 ppm that characterize the aromatic rings of curcumin in the ^1^H-NMR spectra; this signal indicates the remaining chemically stable curcumin. The intensity of the pure curcumin’s characteristic absorption bands at 1627 and 1600 cm^−1^ was significantly reduced. It could occur by the curcumin encapsulation in the cyclodextrin complex. Likewise, the intensity at the 1627 cm^−1^ band and a weakened signal of the bands associated with an enolic group in the inter-ring chain suggested the curcumin’s isomerization. The isomerization of curcumin in the keto–enol may increase the curcumin’s bioavailability and biological activity.

On the other hand, autoclaving sterilization is not advisable for polymeric nanoparticles, because it can increase the hydrolysis reactions and produce a size increase, aggregation, flocculation, or acceleration of Ostwald ripening. Thus, a protective agent such as a tonicity agent, surfactants, or lipid molecules must be added to polymeric nanoformulations [9,36]. In this respect, Sommerfeld et al. [37] evaluated the effect of autoclaving poly butyl cyanoacrylate nanoparticles with different stabilizers: Dextran, Poloxamer, and Polysorbate. The autoclaving of acidic poloxamer suspensions was possible, and the particle size did not reveal a significant increase (≈300 nm). Contrariwise, the polysorbate nanoparticles agglomerated, and they formed nonsuspendable sediment. Similar methacrylate-based nanoparticles to transport DNA were designed by Boss et al. [19]. Those particles presented aggregation and lost transfection efficiency after autoclaving. Furthermore, the polyplex’s chemical and physical characteristics changed during autoclaving, in consequence, damaging the DNA cargo.

Nowadays, companies such as Otonomy Inc. already have patented otic formulations sterilized by autoclaving [38]. They declare terminal sterilization via autoclaving without losing the active agent excipients or polymeric components in the published document. Furthermore, they reported an improved in vitro release of the active agent after the autoclaving sterilization of the micronized formulation. Likewise, Advanced Magnetics Inc. (AMAG) patented a formulation of iron oxide nanoparticles as an imaging tool for liver lesions, which are sterilized by autoclaving [39]. Similarly, Northwestern University is the owner of a patent of citrate-stabilized nanoparticles treated with diethylpyrocarbonate and autoclaving [40]. The nanoparticles are then capped with a double-stranded RNA sequence to enhance the knockdown activity inside the cells. On the other hand, in the patent of Amphastar Pharmaceuticals Inc., they tested a heating period around 100 °C combined with a normal autoclaving cycle in liposomes based on soybean oil and egg lecithin to entrap propofol [41]. The liposomes exhibited a retarded growth of *Pseudomona aeruginosa* in inoculated samples of the formulation.

### 3.3. Advantages and Disadvantages

The main advantage of autoclaving is that it is a simple process that can be easily performed without expensive materials or equipment. Moreover, the simplicity of the technique allows a quick analysis of the autoclaving effects on nanoparticle systems. Interestingly, the extreme autoclaving conditions represent an opportunity to obtain nanoparticles and the sterilized system simultaneously [35,42]. Selvi et al. [22] fabricated silver nanoparticles by autoclaving a *Canna indica* L. aqueous rhizome extract and AgNO_3_. They found a well-defined characteristic of the typical silver nanoparticles in a size range from 40 to 80 nm. The silver nanoparticles displayed excellent antimicrobial activity.

However, after autoclaving, the biological residues such as lipopolysaccharides could be present and induce a proinflammatory immune response after administering the particles. Hence, it is essential to execute all the necessary analyses, such as the pyrogen test, after the sterilization process [43].

On the other hand, the increasing temperature followed by cooling could change the nanoparticles’ physical and chemical properties in some instances (Table 1). Nonetheless, the effect of autoclaving in nanoparticle systems depends on the nanoparticles’ base materials. For example, metal nanoparticles do not exhibit significant physicochemical changes after autoclaving but may present aggregation, which can be improved using stabilizers and nanoparticle capping techniques [11,28,30,31,32]. In lipid nanoparticles, adequate surfactants can protect the lipidic core and keep the nanoparticle properties after moist heating exposure. Using a surfactant with a high cloud point, where the molecule is likely to dissociate from the particle, is a determinant to preserve the nanoparticle properties after autoclaving [44]. On the other hand, polymeric nanoparticles generally present low glass transition points, and the thermal treatment can increase hydrolysis reactions and produce size increases, aggregation, flocculation, or the acceleration of Ostwald ripening [37].

Finally, autoclaving could also induce modifications in the drug or biological content into the nanoparticles, leading to loss of their therapeutic activity or side effects [7,43,44,45]. Thus, it is crucial to select no thermolabile nanoparticles or drugs to achieve a sterile carrier by autoclaving.

In summary, the possibility of autoclaving nanoparticle formulations without changing their chemical and physical properties is still a challenge. Therefore, identifying drugs and materials that maintain their chemical properties after autoclaving will allow the design of more effective and innocuous nanoparticles to carry effective therapeutic molecules.

**Table 1:** Summary of the effects of sterilization by autoclaving on nanoparticle systems.

Nanoparticle	Nanoparticle Size (nm)	Autoclaving Conditions	Effect of Sterilization on Nanoparticle	Ref.
Gold Nanoparticles capped with PEG or Tiopronin	2–60	134 °C/40 min	The PEG shell had better chemical stability around metal cores after autoclaving than the tiopronin shell.	[8]
Citrate-stabilized Silver nanoparticles	20–80	121 °C/30 min	No changes in particle integrity and hemocompatibility were found. Nanoparticles did not vary their sizes after autoclaving.	[30]
Dextran-coated magnetic iron oxide nanoparticles	131.6	121 °C/20 min	The dextran shell did not undergo alteration or destruction. No significant difference in mean sizes was detected. No apparent influences from autoclaving on nanoparticles magnetic behavior were found.	[11]
ZnO and mesoporous silica-ZnO nanoparticles	5–20	---	Autoclaving plus ultrasound stimulation decreased the bacterial concentration of the nanoparticles.	[32]
Silver nanoparticles	40–80	121 °C/15 min	Nanoparticles presented a typical X-ray diffraction pattern for silver nanoparticles.	[42]
Trialurine and phospholipids	200–300	121 °C/20 min	Particle size and Z potential were stable. A slight reduction of the incorporated drug was detected, probably due to drug hydrolysis and the formation of a drug’s hydrophilic form.	[46]
Trimyristin, tripalmitin or Tristearin, with soy lecithin, poloxamer 188, and stearylamine	60–170	121 °C/20 min	Sizes presented increases, and the Z potential changed to positive. The EE did not significantly change. SLNs stabilized with polymer presented a partial collapse of surface adsorbed polymer and particle aggregation.	[13]
Compritol 888ATO, Poloxamer 188	200–250	121 °C/15 min Or 110 °C/30 min.	The particle size increased, whereas Z potential decreased from −16.9 ± 0.7 to −20.5 ± 0.5. The size increase was attributed to a distortion of the mechanical properties of the surfactant film.	[47]
Compritol 888ATO, Poloxamer 188, Tween 80, glycerin	149	110 °C/30 min	The size, Z potential, pH, and EE did not significantly change.	[10]
Liposomes DPPC/DPPG EPC/EPG	200	121 °C/15 min. N_2_ presence.	The particle size of the liposome did not change. Liposomes prepared at pH 7.4 presented a slight change in the gel-sol transition.	[48]
PCL with Cremophor RH40, Synperonics, Tonc P787, or MPS.	130–230	121 °C/20 min	Nanoparticles stabilized with cremophor RH40 presented massive aggregation. A decrease of the pH was detected in all preparations, probably by the oxidation of the surfactants.	[9]
Polybutylcyanoacrylate. Dextran, Poloxamer or Polysorbate	200–300	121 °C/20 min	Particle sizes did not show a significant difference. Dextran nanoparticles did not show an increased particle size, but the size increased without cooling. The polysorbate nanoparticles agglomerated in scarcely suspendable sediment.	[37]
Chitosan-carboximethyl dextran	538	121 °C/30 min	Sizes presented a decrease. No apparent changes in the structure of the polymer.	[49]
PEGylated poly (y-benzyl-l-glutamate)	120	121 °C/20 min	Increased nanoparticle size and polydispersity index accompanied with massive aggregation and precipitation.	[15]
PEG-b-polycaprolactone.	45–105	121 °C/20 min	The presence of medium-chain triglycerides reduced drug leakage in the sterilization process. The drug loading content did not present a significant reduction	[36]
Hydroxyapatite nanoparticles	100	120 °C/20 min	Nanoparticles did not present chemical structure alterations. Nanoparticles synthesized by the wet chemical method showed agglomeration.	[34]
Curcumin-Hydroxypropyl-β-cyclodextrin complex and curcumin-Sulfobutylether-β-cyclodextrin) complex.	200–300	121 °C/30 min steaming phase followed by a 30 min drying phase.	The cyclodextrin complex could entrap the curcumin efficiently. 1H-NMR spectra indicated chemically stable curcumin. Possible isomerization of the curcumin was detected in the Raman spectra after the sterilization-synthesis process.	[35]
Amphiphilic β-cyclodextrin	170	121 °C/20 min	Particle size and polydispersity showed increases. Nanoparticles exhibited aggregation at the autoclaving temperature.	[24]

Abbreviations: PEG: poly(ethylene glycol), PLGA: poly-(d,l-lactic-co-glycolic) acid, PCL: poly-(ε-caprolactone), and EE: entrapment efficiency.

## 4. Nonionizing Radiation

The two most important nonionizing radiations are infrared (IR) and ultraviolet (UV) radiations. Near-IR radiation (700 to 1000 nm) is usually employed in photothermal materials to convert radiation into heat and kill bacteria; this method is suitable for biological applications due to the high penetration into tissues, presenting a few side effects in the organs [50,51]. However, UV radiation is the most known and applied as a sterilization technique for scaffolds and nanoparticles with biomedical applications; for this reason, the following sections will focus only on this technique.

### 4.1. Fundament

UV radiation is part of the electromagnetic spectrum, and it comprises wavelengths in the range from 40 to 400 nm. This radiation is divided into four regions: Vacuum UV (40–200 nm), UV C (200–280 nm), UV B (280–315 nm), and UV A (315–400 nm) [52,53]. This radiation produces atoms’ excitation, promoting electrons within molecules’ atomic orbitals. This excitation induces damage to DNA molecules, which prevents DNA replication and inactivates the microorganisms. UV radiation is widely used in the disinfection and sterilization processes of material surfaces and transparent biodegradable scaffolds. In this context, two crucial parameters that must be monitored are the exposition time and the specific wavelength of employed UV irradiation. Furthermore, the interaction of the UV radiation and the material nature should be analyzed, especially in biomedical applications, due to possible changes in the surface structure, morphology, hydrophilicity, and thermal stability induced by short-wave radiation [54].

### 4.2. Applications

Numerous research groups have explored the effects of irradiation in materials employed in the nanoparticle’s elaboration, such as polyamides, polymethylmethacrylate, chitosan, starch, and pectin [55,56,57] (Table 2).

In this regard, Borcia et al. [58] reported that, after five min of UV irradiation, polymers such as polyamide-6 (PA-6) and polytetrafluorethylene presented lower changes in hydrophilicity and polarity compared with other irradiation processes. The authors concluded that the exposure duration selected could be much higher for the UV component. Similarly, changes in polymethylmethacrylate due to UV irradiation were also studied [59]. The authors reported polymer surface damage, which results in a decrement in strength and the Young’s modulus. Recently, in a sophisticated study, Kowalonek [60] analyzed the modification of chitosan–pectin complexes at different UV radiation times. The morphology and thermal stability of the complexes were not affected by the irradiation; however, an oxidative degradation was observed, resulting in more hydrophilic surfaces after UV treatments.

Changes induced by this sterilization technique in metallic and polymeric nanoparticles have also been reported. For instance, gold nanoparticles with two different sizes and coatings (Au@tiopronin and Au@PEG) were irradiated with UV for 12 h [8]. The obtained micrographs, through TEM, revealed that Au@tiopronin nanoparticles presented agglomeration after irradiation, generating a more oversized and irregularly shaped particle. In contrast, changes in the structure of Au@PEG nanoparticles were not detectable. Furthermore, both types of nanoparticles were characterized by a thermogravimetric analysis (TGA) and Fourier-transform infrared spectroscopy (FTIR) without the presence of noticeable alterations. Likewise, Li et al. [11] developed dextran-coated iron oxide nanoparticles and evaluated their changes after 12 h of UV irradiation exposure. The authors demonstrated that the nanoparticles’ magnetic properties and core size were not affected by the irradiation; moreover, the treatment did not induce additional cell toxicity. Similar results were reported by Dutz et al. [23], who revealed that protein-coated magnetic nanoparticles did not exhibit any critical integrity change after 240 min of UV irradiation.

Sodium alginate (SD), another natural material with several biomedical field applications, has also been employed in drug delivery nanosystems [61,62]. Lately, Chansoria et al. [63] evaluated the modifications in the mechanical, biological, and chemical properties generated by UV irradiation in SD. In their study, the irradiation took place in a solid state. SD powder was exposed to 250-nm UV light for one hour, demonstrating that these conditions were adequate versus inoculated samples of *Enterococcus faecalis* and *Escherichia coli* (less than 10^6^ colony-forming units (CFU)/mL). The results also revealed that UV radiation affected neither the molecular weight nor the viscosity of SD. Remarkably, the metabolic activity of adipose-derived stem cells in culture with SD sterilized by UV was the lowest compared with the other sterilization techniques.

Similarly, in 2020, Tapia-Guerrero et al. [64] performed a concise investigation about the modifications triggered by UV radiation on two different polymeric nanosystems. Nanoparticles based on PCL and poly-(d,l-lactic-co-glycolic) acid (PLGA) stabilized by PVA were obtained by the emulsion–diffusion method. Both systems were sterilized by UV irradiation during different exposure times (0.5, 1.0, 1.5, 2, 2.5, and 3 h). The sterilization method was validated inoculating the nanosystems with an independent suspensions of *Escherichia coli*, *Staphylococcus aureus*, and *Candida albicans*; no bacterial growth was observed in any of the evaluated conditions. Based on the FTIR, TGA, and SEM tests, the authors concluded that nanoparticles’ detectable changes were not found after UV radiation.

Besides the sterilization properties, UV irradiation also could be applied as an activator of some nanoparticles, increasing their bactericidal efficacy. In this regard, the photoactivation of TiO_2_ nanoparticles has been reported [65]. The results suggested that photoactivated and nonphotoactivated nanoparticles presented antimicrobial inhibitory effects on pathogenic bacteria (*Staphylococcus aureus*, *Escherichia coli*, and *Bacillus cereus*) in a dose-dependent way. Notably, before the nanoparticle’s evaluation, the authors analyzed UV A exposure in microorganisms, observing that the radiation had minimal effects on the bacterial growth. The authors mentioned that only intense irradiations, such as UV B and UV C, have a disinfecting impact on bacteria; nevertheless, the photoactivation using UV A was enough to observe a higher antimicrobial effect in nanoparticles.

In a similar approach, Yang et al. analyzed the effectivity of the photocatalytic sterilization of TiO_2_ on *Escherichia coli*, *Staphylococcus aureus*, and *Candida albicans* [66]. In this case, the photocatalysis was carried out under UV irradiation at 254 nm, which corresponds to UV C. The irradiation was also focused directly on the microbial suspensions containing the nanoparticles. The combined effect of TiO_2_ nanoparticles and UV treatment resulted in a 100% sterilization efficiency for the G+ and G- microorganisms and 97% for *Candida albicans*. On the other hand, the UV irradiation directly incised to the strains (without nanoparticles) showed a lower potency versus microorganism strains. Likewise, in the absence of UV irradiation, the nanoparticles presented a poor microbe elimination.

Remarkably, despite its extensive utilization in research and development, to our knowledge, there is no available information about nanoformulations patented or approved by the Food and Drug Administration that employ UV radiation for terminal sterilization.

**Table 2:** Summary of the effects of sterilization by UV radiation on the nanoparticle systems.

Time of Exposition	Nanoparticle Type	Loaded Drug	Effect of Sterilization on Nanoparticle	Ref.
30 min	PEG-PLGA	Curcumin	No effect reported	[67]
30 min	PLGA	C-glycosylflavonoid enriched fraction of *Cecropia glaziovii*	No effect reported	[68]
45 min	PEG-AuNRs	-	The absence of bacterial colonies was verified after the culture onto agar plates	[69]
1 h	Chitosan coated magnetic SLN	Letrozole	No effect reported	[70]
12 h	Au@tiopronin NPs and Au@PEG NPs	-	No detectable changes observed	[8]
12 h	Dextran-coated iron oxide NPs	-	No detectable changes observed	[11]
2 h	PCL/PVA and PLGA/PVA	-	No detectable changes observed	[64]
15 min	PLGA-PEG NPs	-	No effect reported	[71]
3 h	Ag NPs and Au NPs	-	No effect reported	[72]

Abbreviations: PEG: poly(ethyleneglycol), PLGA: poly-(d,l-lactic-co-glycolic) acid, and PCL: poly-(ε-caprolactone).

### 4.3. Advantages and Disadvantages

UV radiation is an inexpensive, easy-to-operate, and green technology that allows rapidly obtaining sterile superficies. However, the bacterial sensitivity to UV radiation is altered by several factors, such as medium pH and bacterial growth phase. For example, UV irradiation quickly destroys vegetative bacteria but is poorly effective versus bacterial spores. In viruses, the enveloped ones present less resistance to UV radiation than naked viruses [73]. Besides, UV radiation has a low penetrating power through solids [74].

As observed, UV radiation is a comprehensive sterilization technique applied in the biomedical field, especially in nanosystem elaboration. However, information about the changes in nanomaterials after this process in many investigations is missing. In some cases, the authors did not mention the wavelength employed or even the sterilization technique applied.

## 5. Ionizing Radiation

### 5.1. Fundament

Ionizing radiation is the energy emitted by electromagnetic waves or photons; some examples include gamma rays, X-rays, and electron beam radiation [75]. The ionizing radiation has sufficient energy to dislodge electrons from atoms and molecules and convert them into electrically charged particles called ions. Gamma rays are emitted from radioactive forms of the element cobalt (Cobalt-60) or the element cesium (Cesium-137). Cobalt-60 (^60^Co_27_) decays (disintegrates) into a stable (nonradioactive) nickel isotope (^60^Ni_28_), principally emitting one negative beta particle (of a maximum energy of 0.313 MeV) with a half-life of about 5.27 years. The resulting Nickel-60 is in an excited state, and it immediately emits two photons of energy of 1.17 MeV and 1.33 MeV in succession to reach its stable state. These two gamma ray photons are responsible for radiation processing in the ^60^Co gamma irradiators. X-rays are produced by reflecting a high-energy stream of electrons of a target substance (usually, one of the heavy metals) into the product. Electron beam (or e-beam) is similar to X-rays and is a stream of high-energy electrons propelled from an electron accelerator into products [76,77]. The high energy provided by ionizing radiation produces significant damage when it is absorbed by microorganisms, causing microbial death primarily through the direct energy deposition (ionization) on vital components, such as DNA or enzymes, and disruption of the microbial membranes via free-radical formation [78,79]. Therefore ionizing radiation, also called the “cold process”, has been used to sterilize pharmaceutical application materials, medical devices, pharmaceutical dosage forms, and raw materials such as drugs and excipients. Moreover, it is a method especially suitable for drugs or heat-sensitive drug carriers. For the sterilization of medical devices and pharmaceutical products, a 25-kGy dose is usually recommended, with no further need to provide any biological validation [80,81].

### 5.2. Applications

In the sterilization of polymeric nanoparticles (Table 3), it is important to consider the type of polymer used before being subjected to the ionizing radiation, because polymers such as cellulosic esters, fluorinated ethylene propylene, and polyacetals have poor or fair radiation stability. This instability is appreciated mainly through the presence of crosslinking and chain scission (radiolytic and oxidative); for example, poly(ethylene oxide) and poly(2-ethyl-2-oxazoline) crosslink rapidly [82]. These alterations can occur even at low radiation doses—for example, 4 kGy [80]. On the other side, there are also polymeric materials that exhibit high radiation stability up to 4000 kGy, including polystyrene, poly(*N*-(2-hydroxypropyl)methacrylamide), polysulphone, aromatic polyurethanes, and poly(*N*-vinyl-2-pyrrolidone) [76,82]. Other chemical changes in the polymers caused by ionizing radiation include the formation of gases, low molecular weight radiolysis products, and unsaturated bonds. Likewise, in the presence of oxygen, polymer oxidation leads to the formation of peroxide, alcohol, CO, CO_2_, and trace amounts of various oxygen-containing low molecular weight compounds. Free radicals created by irradiation may remain trapped in the polymer, causing postirradiation “aging” and a decrease in polymer molecular weight if the dose of gamma radiation increases [80,83]. Associated with that, the use of gamma radiation in sterilization during the development of nanoparticles by using proteins or incorporating them as active pharmaceutical ingredients can have a negative and significant effect. The damage or inactivation of proteins results from two different mechanisms. First, gamma ray irradiation directly ruptures the covalent bonds in target protein molecules due to photons depositing energy into the molecules. Second, gamma ray irradiation produces free radicals and other nonradical reactive oxygen species (ROS) that are, in turn, responsible for the majority of protein damage [84]. Nonetheless, the addition of protection agents, such as amino acids or antioxidants, allows for the successful sterilization of protein products. Furthermore, sterilizing the protein in a dry state, such as lyophilized or spray-dried, helps maintain its function [12]. Another important aspect associated with the change in the polymer’s molecular weight caused by radiation is considering the active ingredient structure, as demonstrated by Maksimenko et al. [85]. In that study, the poly (butyl cyanoacrylate) nanoparticles’ molecular weight did not show any significant change, though possibly the presence of doxorubicin contributed to the formulation’s radiolytic stability. This finding suggests that the sterilization parameters for other poly (butyl cyanoacrylate)-based drugs may need to be adjusted, depending on the active ingredient structure.

Due to the terminal sterilization requirement where possible, gamma sterilization has proven to be an effective method, as indicated by its acceptance in the European Pharmacopeia and the United States Pharmacopeia [86]. In industrial production, there are some ionizing radiation-sterilized products based on nanoparticles. For example, Nanobiotix produces Hensify^®^ NBTXR3, a 50-nm nanoparticle composed of crystalline hafnium oxide (HfO_2_) and functionalized by a negatively charged phosphate coating [87]. That product has the European market approval for the treatment of locally advanced soft tissue sarcoma. On the other side, Z-Medica produces QuikClot^®^, a medical gauze containing aluminosilicate nanoparticles that help blood clot faster in open wounds [88]. Both products use gamma rays for terminal sterilization.

**Table 3:** Summary of the effects of sterilization by ionizing radiation on nanoparticles.

Type of Nanoparticle	Nanoparticle Size (nm) before Irradiation	Radiation Conditions	Effect of Sterilization on Nanoparticle	Ref.
Chitosan hydrogel nanoparticles	288 ± 15	Gamma irradiation (cobalt-60 at doses of 8, 13, and 25 kGy)	Formation of visible sediments, even at the lower doses. Considerable effects on the particle size, PDI, and zeta potential compared to control samples for all irradiation doses suggesting nanoparticles’ severe degradation. The presence of sugars such as mannitol and glucose avoided the formation of aggregates. No bacterial growth was observed in the irradiated samples, in all tested conditions, and for all loads of microorganisms.	[14]
Doxorubicin-loaded poly(butyl cyanoacrylate) nanoparticles	245 ± 83	Gamma irradiation (cobalt-60 with a dose rate of 0.9–1.0 kGy/s) and electron beams irradiation (linear electron accelerator with doses of 10, 15, 25, and 35 kGy)	Both irradiations did not influence the mean particle size, with no tendency for particle agglomeration or sedimentation. Doses up to 25 kGy did not lead to any considerable change in the polymer’s molecular mass distribution. An increase of the molecular mass and polydispersity was found in the drug-loaded nanoparticles after irradiation with 35 kGy. The dose of 15 kGy delivered by either gamma irradiation or electron beam irradiation appeared sufficient for the terminal Sterilization.	[85]
Diclofenac sodium loaded- *N*-trimethyl chitosan nanoparticles	129.3 ± 3.8	Gamma irradiation (cobalt-60 at doses of 5, 10, 20, and 25 kGy)	Particle size, PDI, and zeta potential of non-irradiated and irradiated nanoparticles were not statistically different for all irradiation doses. Gamma irradiation did not cause alteration in the chemical properties of sodium diclofenac entrapped in the nanoparticles. The optimum dose for the sterilization of nanoparticles was 10 kGy.	[89]
Silver nanoparticles	20–80	Gamma irradiation (cobalt-60 at doses of 15, 25, and 50 kGy)	The samples were too polydisperse after gamma irradiation at any of the three-dose levels. Most particles appeared to lose their faceted crystalline structure and formed a combination of much smaller particulates and large irregular-shaped aggregates.	[30]
Lyophilised oligodeoxynucleotide-loaded gelatin nanoparticles	200–280	Gamma irradiation (cobalt-60 with a dose of 25 kGy)	Gamma rays did not change the lyophilizes’ visual appearance and did not induce the particles’ collapse. Irradiation had hardly any impact on particle sizes and PDI values. The irradiation was established as a suitable sterilization method of sugar-based and amino acid-based formulations nanoparticles	[12,90]
Papain nanoparticles	7.7 ± 0.9	Gamma irradiation (cobalt-60 with a dose of 10 kGy)	Gamma radiation produced protein crosslinking. The nanoparticles exhibited size control and preserved bioactivity after irradiation. The radiation technique effectively promoted the simultaneous intramolecular crosslinking and sterilization of papain nanoparticles in one step.	[91]
Poly-ε-caprolactone and poly(d,l-lactide-co-glycolide) nanoparticles	228.8 ± 11.60 and 243.1 ± 3.06 respectively.	Gamma irradiation (cobalt-60 at doses of 5 and 10 kGy)	Irradiation at both doses slightly modified the mean particle size and zeta potential but not chemical properties. At 5 kGy of radiation doses, the presence of trehalose as cryoprotectant reduced the cell damage with high concentrations of nanoparticles, but this did not occur at 10 kGy.	[64]
Ciprofloxacin HCl-loaded poly(d,l-lactide-glycolide) nanoparticles	226.1 ± 1.30	Gamma irradiation (cobalt-60 with a dose of 25 kGy)	No significant differences in the particle size and the gamma-sterilized formulations’ zeta potential value compared with non-sterilized samples. Reconstitution after gamma-sterilization appeared to be more difficult than before sterilization for all formulations studied.	[92]

### 5.3. Advantages and Disadvantages

One of the advantages of applying ionizing radiation is the possibility of using it as a sterilization technique while simultaneously carrying out the synthesis of metallic nanoparticles such as gold nanoparticles. This process would eliminate pathogenic or contaminating microbes while promoting nanoparticle formation in parallel, resulting in a quick, cost-effective, and straightforward process [75]. Furthermore, there would be no need for a crosslinking agent for the polymeric materials, rendering such systems free of impurities and potentially toxic residuals, making it an excellent choice for biological and human health applications [93]. Using ionizing radiation in the terminal sterilization process is advantageous, because the gamma rays may penetrate up to 300 mm into materials due to their mass absence, facilitating their extensive use in processing larger product quantities at once. Moreover, these attributes allow employing at final packaging, avoiding further contamination and excessive handling [75,94]. However, this technique’s main disadvantage is the difficulty of gaining access to gamma irradiators. Furthermore, some materials used as capping or stabilizing agents can be damaged by high-energy irradiation and not work effectively. In contrast, X-rays are more accessible radiation sources with a strong penetration; thus, they have attracted considerable attention over recent years [75]. Finally, electron beam irradiation is still a minority of worldwide sterilization; however, there is increasing interest in its use. Despite its low penetration rate, electron beam irradiation sterilizes instantaneously, with a high dose delivery [80].

## 6. Challenges in Choosing the Sterilization Method

**Figure 2:**
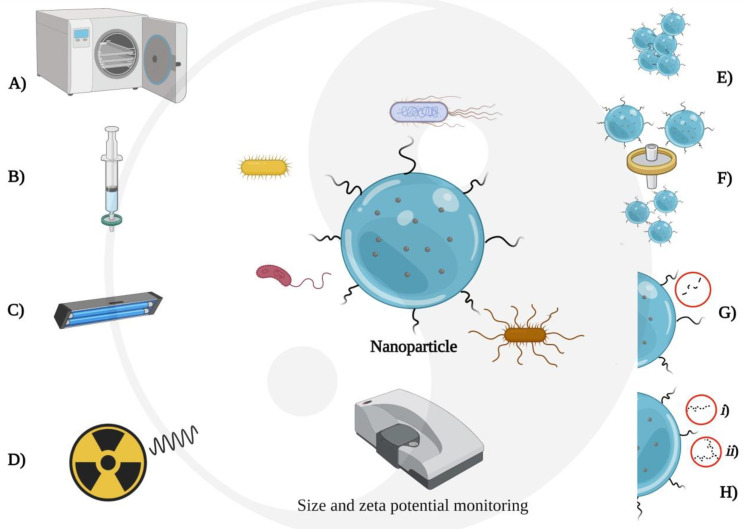
Possible adverse effects of nanoparticle sterilization. Standard sterilization methods: (**A**) autoclaving, (**B**) sterile filtration, (**C**) UV radiation, and (**D**) gamma radiation. Possible adverse effects on nanoparticles for biomedical use: (**E**) aggregation of nanoparticles after autoclaving, (**F**) segmentation of the particle size distribution after filtering, (**G**) possible homolysis of chromophoric groups on the surface of the nanoparticles after UV radiation, and (**H**) homolysis of stabilizing agents influenced by the presence of free radicals from the water radiolysis (**i**) and crosslinking of homolysis products (**ii**). Created with BioRender.com.

As seen above, each sterilization method possesses advantages and drawbacks; thus, the sterilization method’s choice will depend on the formulation’s characteristics, factors associated with batch volume, available methods, and terminal sterilization limitation (Figure 2).

### 6.1. Factors Related to the Formulation

The physicochemical properties of the nanoparticle constituent materials will determine the viability of being subjected to sterilization by a particular method. It will also depend on the type of nanoparticle, the material complexity involved, and their proportion and physical state, in addition to considering the susceptibility of the drug [7]. Nanoparticles with a size distribution smaller than 200 nm, a low viscosity, and a low solid concentration can be sterilized by filtration. Materials with low melting points are conveniently directed towards radiation methods, while nonionizing radiation processes with poor sterilization quality can be supplied with ionizing radiation.

The sterilization process’s impact can affect the architecture of the nanoparticle from the surface to the core. For example, the autoclaving process will supply energy to the system, which can cause the release of stabilizers (desorption) [9]. The desorption level will be a function of external factors such as pH, ionic strength, temperature, energy supplied, interaction of the stabilizer with the matrix material, and its chemical nature to withstand high temperatures. Consequently, autoclaving may lead to decreased stability [24], zeta potential changes; and a slight modification of the average particle size; size distribution, and PDI value [37]. In some matrix polymers, the glass transition temperature (Tg) can be overcome and affect the drug entrapment capacity.

The UV radiation would preferably affect the stabilizing materials located on the surfaces of nanoparticles [58]. The homolysis of the backbone in the formation of free radicals would also produce the nanoparticles’ loss of stability in an aqueous medium; therefore, a change could be observed in the average particle size, size distribution, PDI value, and, significantly, in the value of the zeta potential. The formation of free radicals can affect all the biological applications of the nanoparticles. Changes in the matrix polymers would be less significant, although it depends on the amount of energy supplied and the nanoparticle’s composition [64]. Concerning this, FTIR could evidence changes in the stabilizer’s backbone. In a controlled case study, only the stabilizer could be affected by UV, and the corresponding physicochemical characterization could be carried out. In another case study, a dose-response curve can be used to delimit the universe of study concerning the sterilization method and the guarantee that there will be no structural alterations and toxicity.

The gamma radiation process is par excellence a high energy transfer with a high capacity for permeation in materials [95]. The operating range in kGy emission is limited for each material and type of nanoparticle. When the energy supplied is higher than that necessary to sterilize, the undesirable side effects produced by sterilization by gamma radiation are similar to UV but with a greater magnitude [64]. Materials external to the nanoparticle are more susceptible to modifications. The stabilizer can undergo homolysis from chromophoric groups’ reactions and by radiolysis of the water; then, the multiple species of free radicals generated could excise the backbone. There is a greater probability that the nanoparticle’s matrix polymer can be affected, which can be demonstrated with a change in the molecular weight, entrapment capacity, and release profile [85]. Noticeably, the parameters of the average particle size, type of size distribution, PDI value, and zeta potential will also be modified. In conditions of higher energy applications, extreme conditions that far exceed sterilization; not only can there be homolysis, a different crosslinking can also be induced in the materials [91], generating novel chemical species [96].

On the other hand, terminal sterilization can also be applied to lyophilized products, minimizing the absorbing effect of heat or radiation by water on the materials. The presence of cryoprotectants in lyophilized products can also protect against radiation phenomena and minimize the possible changes in the nanoparticles’ physical, chemical, and biological effects [64].

Preformulation studies aimed at evaluating the changes in nanoparticles loaded with a drug, empty nanoparticles, and individual materials will help prevent possible drastic changes. The analysis of the materials separately and their biological correlation could help to elucidate the possible specific affectations caused by the sterilization method.

### 6.2. Issues Related to Batch Volume

Small volume batches can be effortlessly processed, regardless of the sterilization method of interest. However, when increasing the volume (for example, 10 L or more), it may vary the cost of sterilization, the time, and the method’s effectiveness. The batch can be fractionated in autoclaving, although large volume containers’ energy consumption will be considerable, while filtration systems for large volumes require more expensive accessories, and the flow capacity can be a limitation to consider in the manufacturing time. From this perspective, the nonionizing radiation system would seem like an option over the other methods; nevertheless, it has the limitation of a surface effect, while the large volume containers would further restrict the effect of UV radiation. Gamma radiation is an alternative that, under validated conditions, can be efficient in large volume containers. The variation in cost and time due to the increase in volume is less than in the other methods, facilitating its use at an industrial level.

### 6.3. Aspects Related to Available Methods

Except for gamma radiation and sources of radioactive elements, the other sterilization methods are universally accessible. In all countries, access to sterilization services for gamma radiation sources involves a request with a detailed and meticulous review for the type of safeguard with high national security. This type of processing and administrative documentation presupposes different logistics in access and transport times, unlike the other sterilization equipment that can be freely arranged within the organization and its laboratories.

### 6.4. The Limitation of Terminal Sterilization

Terminal sterilization is understood as the inactivation of viable microorganisms; it ensures that a product does not have bacterial growth but, possibly, endotoxins. Although the described methods can be highly effective, it is always advisable to guarantee an aseptic production line, because terminal sterilization cannot replace the lack of control in the previous product manufacturer stages [97]. Even though there are various methods of depyrogenation, the high surface–volume ratio of the nanoparticles favors a significant interaction with all the surrounding components and possible adsorption phenomena, which would complicate the complete elimination of the pollutants.

Finally, regardless of the processed material, the chosen method’s maximal conditions do not necessarily have to be used, which would probably compromise the nanoparticles’ properties. It is possible to combine filtration and UV radiation or filtration and gamma radiation with low radiation energy emission to ensure that the materials’ structures will not be altered.

## 7. Conclusions

As mentioned in the previous sections, all sterilization methods require validations for specific nanoparticle samples. Validation will involve biological safety tests, structural characterization, and toxicity monitoring. Due to the complexity in the constitution and proportions of constituents of the different nanoparticles for biomedical use, it is difficult to generalize the operating conditions in energy radiation. Interestingly, the absence of changes in the physical parameters such as particle morphology, particle size, PDI value, or even zeta potential does not necessarily mean that there are no chemical or interaction changes between the materials. It has sometimes been shown that, after applying terminal sterilization, the physical changes are absent, but the presence of toxic phenomena in biological tests is found. Scarcely mentioned, but viable, in samples susceptible to radiation emission changes, combining two methods is possible. For example, it could be used prefiltration to reduce the load of microorganisms and then low UV emission or gamma radiation for the terminal effect. Finally, a terminal sterilization process will not guarantee the total safety of a nanoparticle sample. It is necessary to guarantee the aseptic manufacturing conditions, reagent purity, and follow-up in the product’s different stages.
